# The gut remembers: the long-lasting effect of medication use on the gut microbiome

**DOI:** 10.1128/msystems.01076-25

**Published:** 2025-09-22

**Authors:** Pamela Ferretti

**Affiliations:** 1 Section of Genetic Medicine, Department of Medicine, University of Chicago 2462, Chicago, Illinois, USA; Medical Research Council Toxicology Unit, Cambridge, United Kingdom

**Keywords:** gut microbiome, medication, metagenomics, long-term effects, electronic health records

## Abstract

Growing evidence suggests that antibiotics and many human-targeted medications can alter the gut microbiome composition, but the persistence of these effects remains unclear. In their article, Aasmets and colleagues (O. Aasmets, N. Taba, K. L. Krigu, R. Andreson, et al., mSystems e00541-25, 2025, https://doi.org/10.1128/msystems.00541-25) leveraged electronic health records (EHR) and stool metagenomic data from 2,509 individuals to assess the impact of past medication use (up to 5 years prior to sampling) on the gut microbiome composition. They found that nearly half of the 186 tested drugs had long-term effects, with antibiotics, beta-blockers, benzodiazepine derivatives, proton-pump inhibitors, and antidepressants associated with microbiome changes that persisted for years after intake. For some medications, the effects were additive, with greater impact observed after repeated use. Overall, the authors highlight how medication use in the years preceding sample collection represents an often overlooked confounding factor in microbiome studies and emphasize the utility of combining EHR with microbiome data to assess the impact of past medication use.

## COMMENTARY

Over the past decades, medication use has steadily increased worldwide, with individuals frequently taking multiple medications (polypharmacy), often repeatedly over extended periods of time (1). Yet, our understanding of how these practices impact the gut microbiome, a key component to our health, remains in its infancy (2). A landmark *in vitro* study (3) profiling the effects of over 1,000 marketed drugs on representative human gut bacterial isolates found that 78% of antibiotics and 24% of human-targeted drugs affected commensal bacteria, including keystone species such as butyrate and propionate producers. More recently, another *in vitro* study (4) revealed that beta-blockers (used to treat hypertension), selective serotonin reuptake inhibitors (SSRIs, a class of antidepressants), and statins (used to reduce cholesterol levels) alter bacterial functions relevant to the host’s health, such as vitamin biosynthesis. Metagenomic *in vivo* studies (5–8) have further confirmed that commonly prescribed medications, such as metformin (antidiabetic), proton-pump inhibitors (used to decrease stomach acid production), and statins, can significantly alter the human gut microbiome composition and function, often in a dose-dependent manner. In addition, such medication-induced shifts have been shown to confound disease-associated microbial signatures (7), potentially hindering biomarker discovery. However, far less is known about the persistence of these alterations in the human gut over long periods of time (>1 year).

**Fig 1:**
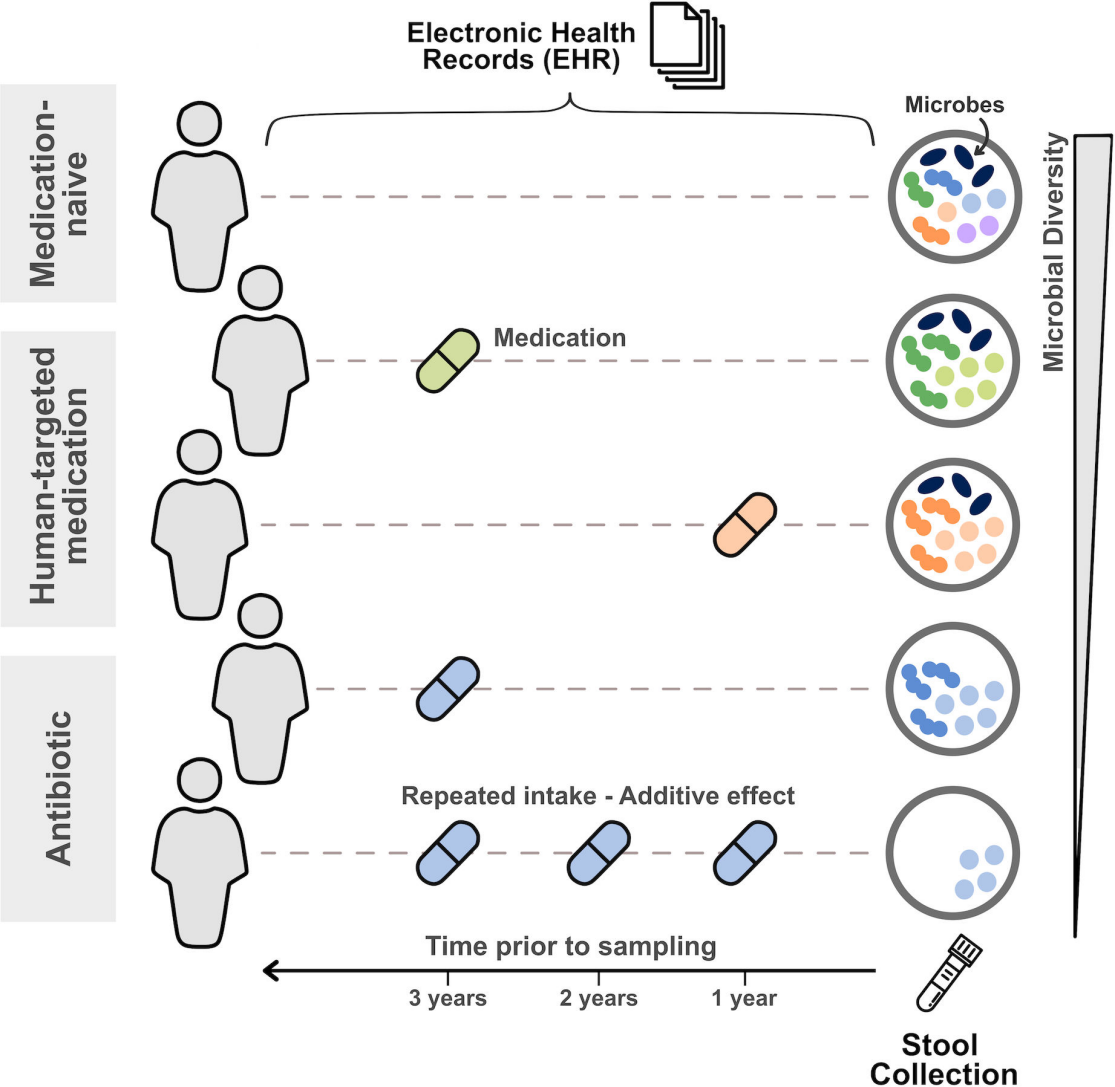
Schematic representation of how medication use in the years prior to sample collection can impact gut microbiome composition.

To explore the long-term impact of medication use on the gut microbiome, Aasmets et al. (9) integrated metagenomic profiling of stool samples from a previously published cohort of 2,509 individuals (10) with detailed past medication data extracted from electronic health records (EHR) (Fig. 1). EHR allowed the authors to characterize participants’ current and past medication use, up to 5 years prior to microbiome sampling.

First, Aasmets and colleagues set out to assess the effects of active medication usage on the gut microbiome. They compared the gut microbiome of individuals actively taking a target medication to those with no history of use of that medication in the previous 5 years, while adjusting for possible confounding factors such as BMI, gender, and age. Remarkably, they found that nearly 90% of the 186 medications tested were associated with changes in overall microbial diversity or shifts in the abundance of specific taxa. Beta-blockers, macrolides, and biguanides were negatively correlated with bacterial diversity and positively correlated with presence and abundance of members of the *Lachnospiraceae* family. While some of these associations have been observed in previous studies (2, 5, 8), disentangling the effects of medication use on the gut microbiome from those of the underlying conditions for which the medications were prescribed remains challenging. The authors also observed that individuals taking a higher number of unique medications at the time of sampling tended to have lower microbial richness, suggesting an additive effect of polypharmacy on microbiome diversity loss. Using machine learning models trained to detect antibiotic use from gut microbiome profiles, the authors were also able to predict the use of several human-targeted medications, suggesting shared microbial signatures between these drug classes. Certain taxa, such as *Dorea longicatena* and *Eubacterium* species, were depleted by antibiotics but not by human-targeted medications.

After identifying drug-microbiome associations during active medication intake, the authors investigated whether those effects persisted long after the medication had been discontinued, indicating potential carryover effects. To do so, they compared individuals who had stopped taking a medication more than 1, 2, 3, or even 4 years before sampling to those with no intake in the prior 5 years. The authors identified potential carryover effects in 42% of the tested drugs. The impact of broad-spectrum antibiotic use (e.g., macrolides and penicillins) and of some human-targeted medications (e.g., benzodiazepine derivatives and antidepressants) could be detected even if the medication was discontinued more than 3 years before sample collection. These results are in line with a recent preprinted study that also found that certain antibiotic-associated microbial signatures were detectable in stool samples for several years after antibiotic intake (11). To further validate long-term drug effects, the authors analyzed microbiome changes in a subset of 328 individuals who provided a second stool sample (T2), roughly 4.4 years after the first sampling. They compared individuals who initiated specific medications between the two time points with medication-naive controls and confirmed the results found in the larger, cross-sectional cohort.

These results clearly indicate that medication use has long-term effects on the gut microbiome. They also highlight the necessity of moving beyond the standard practice in microbiome research of recording only medications used at or shortly before sampling and to include those taken in the years preceding sample collection. Finally, this study showcases the value of accessing EHR, which, unlike self-reported data, provides a less biased and more detailed history of medication use. As the field gains deeper insight into the interactions between microbes and medications, the routine integration of metagenomic data with EHR will improve our interpretation of microbiome results by better accounting for potential confounding factors and will advance our understanding of how medications influence our gut microbiome, and ultimately our health, in unintended ways.
